# A web-based software for group decision with analytic hierarchy process

**DOI:** 10.1016/j.mex.2023.102277

**Published:** 2023-06-30

**Authors:** Rafael Verão Françozo, Luiz Sérgio Velasquez Urquiza Junior, Elana Souza Carrapateira, Bruna Cristine Scarduelli Pacheco, Márcio Teixeira Oliveira, Guilherme Botega Torsoni, Jiyan Yari

**Affiliations:** a Federal Institute of Education Science and Technology of Mato Grosso do Sul, Brazil; b University of Araraquara, Brazil

**Keywords:** Open-source software, Decision support systems, Software for OR, AHP-WEB

## Abstract

The Analytic Hierarchy Process (AHP) is a multi-criteria decision support method and is widely applied in many areas. The original AHP method proposed by Thomas L. Saaty in the 1970s requires (n²-n)/2 comparisons. The number of required comparisons can make using this method challenging for maintaining consistent judgments in problems involving many criteria and/or alternatives. Furthermore, the available software is platform-dependent and generally does not support group decision-making. In this paper, we present software for AHP that demands n-1 comparisons. Additionally, the software supports group decision-making using individual aggregation of priorities with arithmetic and geometric means. The system is available at http://ahpweb.net/ and is accessible from any internet-connected device. It currently has more than 100 users and dozens of decision problems in various areas.

• The original AHP formulation requires (n²-n)/2 comparisons per cluster which makes it difficult to make consistent judgments.
• AHP avaliable software does not enable group decision making.
• The proposed system AHP-WEB fills these gaps. The method demands n-1 comparisons per cluster without any inconsistency and allows group decision making on a web system.

## Graphical abstract

**Figure:**
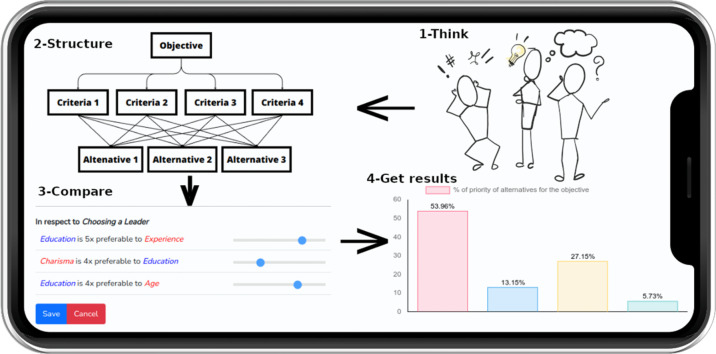

Specifications table

**Table:** 

Subject area:	Computer Science
More specific subject area:	Operations Research, Multicriteria decision making
Name of your method:	AHP-WEB - T. L. Saaty, Decision making with the Analytic Hierarchy Process, International journal of services sciences 1.1 (2008) 83–98, https://doi.org/10.1504/IJSSci.2008.01759.
Name and reference of original method:	Analytic Hierarchy Process
Resource availability:	The source-code are avaliable at https://github.com/rvfrancozo/Simple-AHP-WEB

## Introduction

The Analytic Hierarchy Process (AHP) is one of the most extensively studied and employed methods in applications related to multi-criteria decision-making (MCDM) in fields such as economics, politics, and engineering [[1], [2], [3], [4], [5]]. The AHP was introduced by Thomas L. Saaty [6] and enables decision-makers (DMs) to use multiple criteria in a quantitative way to evaluate and select the optimal alternatives from qualitative judgments [7]. The AHP has been described in many papers according to several related literature reviews [[3], [4], [5]].

The structure of the AHP is similar to a network on a hierarchical level where each vertex is a criterion, sub-criterion, or alternative. Vertices on the same level are compared to each other in relation to each vertex one level above. This task demands k(n²-n)/2 comparisons, where *k* is the number of vertexes (criteria or sub-criteria) one level above, and *n* is the number of vertexes on the same level to be compared to each other. Thus, a decision problem with 9 criteria and 21 alternatives demands 1890 comparisons. Considering that each DM needs 1 min on average for each judgment, the complete process will take more than 31 h [8].

The number of needed judgments could be a challenge for the DM in decision problems with many criteria, sub-criteria, or/and alternatives, especially to maintain consistent judgments [2]. According to Saaty [6], in practice one should reject matrices with values of consistency ratios greater than 0.1 and accept values lower than 0.1. Another issue of the AHP is related to the transitivity. Considering a decision problem with three criteria {A, B, C}, if the relative importance of A is greater than that of B (9x) and the relative importance of B is greater than that of C (9x), then no judgment will be possible, within the limits of the nine-point Saaty scale, between A and C such that the consistency index is less than 0.1 [9].

In addition, the most popular software for the AHP, Super Decisions, MakeItRational, TransparentChoice, Expert Choice, and others is platform-dependent, not open-source, not free, and there is no free version that enables group decision-making.

In the face of the questions presented, this study aims to contribute through a web-based software based in the modified version of AHP called AHP-Express avaliable at [2]. The software enables group decision-making and demands k(n-1) judgments. The remaining judgments are automatically calculated. The DM also can revisit the judgments and consciously make an inconsistent judgment.

## Background

### Analytic hierarchy process fundamentals

**Fig. 1:**
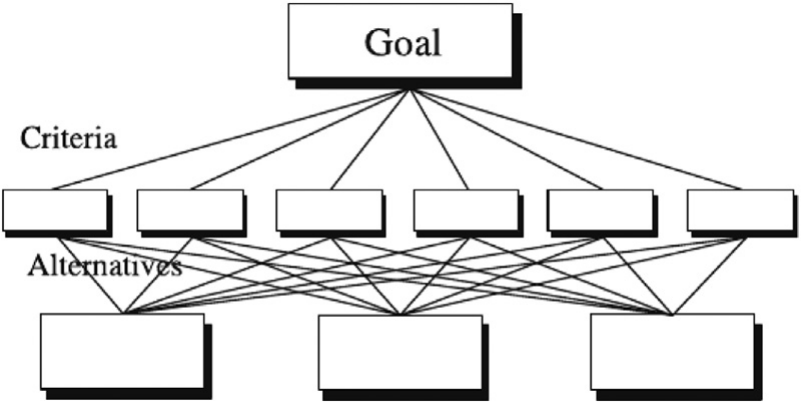
A three level hierarchy. *Source:* Saaty & Vargas, 2012, p.3.

Similar to other multi-criteria decision analysis (MCDA) methods, the AHP provides stepping-stones for finding a compromise solution [10]. The AHP consists of three steps: 1 – hierarchy formation; 2 – pairwise comparisons; and 3 – verification of consistency [7,11]. The first step focuses on the problem structuring in a hierarchical form, where the main goal is on the top of the hierarchy, the second level represents the criteria, and the last the alternatives [10]. The hierarchy can have sub-criteria according to the convenience of the DM or problem requirements. Fig. 1 illustrates this structure.

The second step is to conduct pairwise comparisons between the criteria with respect to the main goal, and between the alternatives with respect to each criterion. This results in a pairwise matrix for each set of criteria/alternatives. The comparisons are made with a numeric scale “that indicates how many times more important or dominant one element is over another element with respect to the criterion or property with respect to which they are compared” [11, p. 85]. DMs express their opinions on pairs using verbal terms, which are then associated to numeric values. The numeric scale is only a way to transform a verbal judgment into a numeric value. Some scales are available in the literature such as the Saaty fundamental scale and balanced scale proposed by [12] as shown in Table 1.

**Table 1:** The Saaty and balanced scales.

*Intensity of Importance*			
*Saaty's scale*	*Balanced scale*	*Definition*	*Explanation*
1	1	Equal Importance	Two activities contribute equally to the objective
2	1.22	Weak or slight	
3	1.5	Moderate importance	Experience and judgment slightly favor one activity over another
4	1.86	Moderate plus	
5	2.33	Strong importance	Experience and judgment strongly favor one activity over another
6	3	Strong plus	
7	4	Very strong or demonstrated importance	One activity is favored very strongly over another; its dominance demonstrated in practice
8	5.67	Very, very strong	
9	9	Extreme importance	The evidence favoring one activity over another is of the highest possible order of affirmation
Reciprocals of above		If activity *i* has one of the above non-zero numbers assigned to it when compared with activity *j*, then *j* has the reciprocal value when compared with *i*	A reasonable assumption
1.1–1.9		If the activities are very close	May be difficult to assign the best value; however, when compared with other contrasting activities, the size of the small numbers would not be too noticeable, but they can still indicate the relative importance of the activities

*Source:* (Pöyhönen et al., 1997; Saaty, 2008, p.85).

Other scales are available in [13]. Regardless of the scale used, a matrix of pairwise comparisons is generated as shown in Expression 1.

(1) $$A=\left(\begin{array}{cccc} 1 & a_{12} & \cdots & a_{1n}\\ \frac{1}{a_{12}} & 1 & \cdots & a_{2n}\\ \vdots & \vdots & \ddots & \vdots \\ \frac{1}{a_{1n}} & \frac{1}{a_{2n}} & \cdots & 1 \end{array}\right)$$

Therefore, a comparison matrix exists for each level of the hierarchy, including from the criteria in respect to the main objective, from (possible) sub-criteria in respect to the upper criteria, and from alternatives in respect to each immediately upper criterion. Once the pairwise comparison matrix is established, the local priority vector is calculated. Many different methods are available to obtain the priority vector.

The eigenvector method is probably the most popular (and cumbersome) and referenced in Saaty's papers and books. In the eigenvector method, the priority vector generated by the pairwise comparison of the elements is obtained by calculating the right eigenvector associated with the maximum eigenvalue of the decision matrix [14]. The eigenvector method demands a decision matrix with good (otherwise perfect) transitivity or consistency.

The geometric mean method is another method widely used to estimate the priority vector. This method is described in [14] and [15]. In the geometric mean method, the *n* elements in each row are multiplied, the *n*th root is taken, and the resulting numbers are normalized as indicated in expression 2.

(2) $$w_{i}={\left(\prod_{j=1}^{n}a_{ij}\right)}^{\frac{1}{n}}\sum_{i=1}^{n}{\left(\prod_{j=1}^{n}a_{ij}\right)}^{\frac{1}{n}}$$

Saaty states that the geometric mean method provides a very good approximation to the exact solution to the problem [14, p. 21].

According to Saaty [14] the exact solution is obtained with power method. The priority vector in the power method is obtained raising the matrix to “arbitrarily” large powers and normalizing the results following the procedure in expression 3.

(3) $$w_{i}=\sum_{j=1}^{n}a_{ij}\sum_{i=1}^{n}\sum_{j=1}^{n}a_{ij}$$

By “arbitrarily” we mean an exponent X whose priority vector has no significant change (third or fourth decimal place) from the resultant of the comparison matrix raised to an exponent *X* + 1. The procedure exposed in expression 3 could be used without raising the comparison matrix. The software proposed in this study uses the power method.

Other methods to obtain the priority vector are presented in expressions 4 and 5.

(4) $$w_{i}={\left(\sum_{i=1}^{n}a_{ij}\right)}^{-1}\sum_{j=1}^{n}{\left(\sum_{i=1}^{n}a_{ij}\right)}^{-1}$$

(5) $$w_{i}=\sum_{j=1}^{n}\left[a_{ij}{\left(\sum_{i=1}^{n}a_{ij}\right)}^{-1}\right]\sum_{i=1}^{n}\sum_{j=1}^{n}\left[a_{ij}{\left(\sum_{i=1}^{n}a_{ij}\right)}^{-1}\right]$$

There are a lot of advantages and disadvantages of the different algorithms that can be used for matrix comparison, a comparative study between these and other methods, including the weighted average and least worst square, is available in [16]. If the comparison matrix is consistent, the priority vectors will be the same in all methods.

The third step is the consistency verification of the judgments. For example, considering three criteria A, B, and C, and a DM says that A is better than B and B is better than C, by the axiom of transitivity; therefore, A is better than C and this is a consistent judgment. However, in AHP, a preference of one criterion or alternative to another is given by a verbal/numeric scale and due to the limitations of experiences and expertise as well as the complexity nature of the decision problem, the pairwise comparison matrix could be inconsistent [17].

In the comparison matrix A, if a_ij_ represents the importance of alternative *i* over alternative *j* and a_jk_ represents the importance of alternative *j* over alternative *k* and a_ik_, the importance of alternative *i* over alternative *k*, must equal a_ij_/a_jk_ or a_ij_/a_jk_ = a_ik_ for the judgments to be consistent [9,14].

The AHP subjective weighting schemes are required by DMs, which could not be “perfectly rational and can precisely express his preferences on all pairs of independent alternatives and criteria using positive real numbers” [9, p. 33]. Therefore, some inconsistency of judgment is tolerated.

To obtain the priority vector, methods to calculate the consistency of judgments include Consistency ratio [14]; Geometric consistency index [15,18]; Harmonic consistency index [19]; Ambiguity index [20]. Here we focus on consistency ratio, expressed in 6, because it was proposed by Saaty and is the most widely used method to obtain the priority vector [21].

(6) $$C\;R\left(A\right)=\frac{CI\left(A\right)}{RI_{n}}$$

When *CR(A)* is the consistency ratio of a comparison matrix A and if its value is greater than 0.1, the judgments should be revisited [14,22]. The *CI(A)* value is the consistency index and is given by:

(7) $$C\;I\left(A\right)=\frac{\lambda_{max}-n}{n-1}$$

Where λ_max_ is the maximum eigenvalue of a pairwise comparison matrix A of order *n*. RI_n_ is the random index – an estimation of the average CI, a real number obtained from a set of randomly generated matrices of size *n* [9,14,22]. The values of RI have been studied for many years. Table 2 shows some of the values presented in the literature.

**Table 2:** RI_n_ values presented in the literature.

*Matrix order*	*Ri_n_ values*				
	*Saaty (1977)*	*Aguarón & Moreno-Jiménez (2003)*	*Alonso & Lamata (2005)*	*Saaty & Vargas (2012)*	*Franek & Kresta (2014)*
3	0.416	0.525	0.5247	0.52	0.525
4	0.851	0.882	0.8816	0.89	0.882
5	1.115	1.115	1.1086	1.11	1.110
6	1.150	1.252	1.2479	1.25	1.250
7	1.345	1.341	1.3417	1.35	1.341
8	1.334	1.404	1.4057	1.40	1.404
9	1.315	1.452	1.4499	1.45	1.451
10	1.420	1.484	1.4854	1.49	1.486
11	1.395	1.513	1.5140		1.514
12	1.482	1.535	1.5365		1.536
13	1.491	1.555	1.5551		1.555
14	1.470	1.570	1.5713		1.570
15	1.466	1.583	1.5838		1.584
16		1.595			

Except for [6], the results are very close. Saaty [6] constructed a sample of size 50, whereas the others used a very large set of randomly generated matrices (up to 500,000). Values of RI from [22] are probably the most used in the literature. While most studies limit IR values for matrices in the order of 15 at most, [23] provide IR values for matrices up to 39. However, assuming a decision problem with 39 criteria and since AHP demands n(n-1)/2 comparisons, only 663 comparisons between the criteria would need to be revised if the inconsistency is greater than 0.1, which is enough to discourage any DM from using AHP!

### Group decision-making

Decision-making in organizations are collective constructions that go through several DMs. The AHP method is applicable to both individual and group decisions. Just like everything else in the AHP, many methods can be used to aggregate the judgments with the simplest one being consensus among the DMs when it comes to making judgments [24]. According to [25], two main methods can be used to accommodate the views and judgments: aggregation of individual judgments (AIJ) and aggregation of individual priorities (AIP). Each can be aggregated by arithmetic or geometric averages defining weights for each decision-maker.

In AIJ, the *n* matrices of each DM are aggregated into one A^G^, and the priority vector is calculated from A^G^ with any of the methods previously described. AIJ requires satisfaction of the reciprocity condition for judgments which implies that the aggregation must be done with geometric mean [20]. In the AIP, individual priority vectors are derived from each pairwise comparison matrix and aggregated with arithmetic or geometric mean [9,25,26].

In both AIJ and AIP, a weight could be defined for each DM; thus, one DM may have a greater relative importance in the decision-making process than others. [9, p. 168] offer expressions for each aggregation way. Weighted geometric mean of judgments (AIJ) is given by:

(8) $$J_{g}\left(k,l\right)=\prod_{i=1}^{n}J_{i}{\left(k,l\right)}^{w_{i}}$$

Where *J_g_(k,l)* refers to the group judgment of the relative importance of factors *k* and *l; J_i_(k, l)* refers to individual i's judgment of the relative importance of factors *k* and *l, w_i_* is the weight of individual DM *i* where the sum of *w_i_* of all DM is equal to *1*.

Weighted geometric mean and arithmetic mean of judgments (AIP) are given, respectively, by:

(9) $$P_{g}\left(A_{j}\right)=\prod_{i=1}^{n}P_{i}{\left(A_{j}\right)}^{w_{i}}$$

(10) $$P_{g}\left(A_{j}\right)=\sum_{i=1}^{n}w_{i}P_{i}\left(A_{j}\right)$$

Where *P_g_(A_j_)* refers to the group priority of alternative *j, P_i_(A_j_)* to individual *i's* priority of alternative *i*.

As presented, the AHP is a robust method, widely used in decision problems and that has multiple variations for obtaining priority vectors, calculating consistency, and aggregating results. Just like other MCDA methods, the AHP requires substantial amounts of computations that can be assisted by decision analysis software [27].

### Software for AHP

Throughout the years, many pieces of software have been introduced and referenced to in the literature to support the use of the AHP. Although there are some free versions, they are generally commercial and platform-dependent software packages. [27, p.29] have identified 14 software packages for AHP. [28] use the AHP to evaluate the software for AHP, and as results five software packages are selected, of which we were able to locate the four described in Table 3.

**Table 3:** Software for AHP.

*Software Name*	*Free Version*	*Platform*	*Source*
MakeItRational	no	Windows	https://www.transparentchoice.com/
Super Decisions	yes	Windows, Mac	https://www.superdecisions.com/
Expert Choice	no	Windows	https://www.expertchoice.com/
Logical Decisions	30 Day Trial	Windows	https://www.logicaldecisionsshop.com/catalog/

All software packages are proprietary and platform-dependent. MakeItRational now is called TransparentChoice. Super Decisions and Logical Decisions have free versions, the first requires its license to be renewed every 6 months, and the second has a 30-day trial version. There is no free version that allows group decision-making and is accessible via the web (platform-independent).

### AHP-related issues

Despite decades of development, some questions remain about the AHP. In this study, we focus specifically on 3: number of comparisons needed; difficulties in establishing consistency of judgments, and unavailability of online software for group decision.

First point in AHP original formulation is the difficulty in working with large problems, the greater the number of criteria and alternatives the greater the time and effort required to perform the pairwise comparisons [29]. If a decision problem has *n* criteria and *m* alternatives, then *(n²-n)/2* *+* *n(m²-m)/2* comparisons are required. If *n* *=* *m* *=* *10,* then 495 comparisons will be needed. If the DM spends one minute for each comparison, then it will take more than 8 h to complete the comparisons. If the judgments are not consistent, they should still be reviewed. This situation limits or discourages the use of the AHP for decision problems involving a significant number of criteria and/or alternatives. The DM's time is usually limited so more agile methods are desired. The number of comparisons required in AHP has been discussed in recent literature, such as [2,30].

Secondly, the greater the number of criteria/alternatives, the greater the difficulty in maintaining consistent judgments because the larger the matrix of comparisons the more inconsistent combinations are possible. In addition it implies that DM's revise their judgments and reassessments of comparison matrices are very time consuming and the DM's may be unwilling or unable to revise their judgments [31,32]. However, even in decision problems with few criteria/alternatives, three for example, it is possible that the decision-maker has difficulty maintaining a consistent judgment within the 0.1 limit defined by Saaty. Taking a decision problem with three criteria A, B, and C, and using Saaty's fundamental scale, we say that: *A* = 9B and *A* = 1/9C. In this case, there is no value within the limits of the Saaty scale for comparing criteria B and C such that the consistency is less than or equal to 0.1 [33]. The best case would be to say that *B* = 1/9C, and the consistency would be about 0.5. This implies that the decision-maker needs to adjust his/her judgment to be adequate for the method, which sounds a bit unwelcome.

Finally, despite the variety of software that implements the AHP, there are few free options, and to the authors’ knowledge, none are simultaneously for group decision, free of charge, and platform-independent. In response to these three issues, this study presents web-based software that significantly reduces the number of judgments needed in a consistent way and allows group decision. Details of the method are presented in the following section.

### Method foundations

In matrix A presented in Equation 1, *a_ij_* expresses the importance of criterion/alternative *i* over the *j*, and *a_jk_* the importance of *j* over *k*. Therefore, the importance of criterion/alternative *i* over *k* is given by *a_ij_a_jk_* = *a_ik_* [22]. Thus, *a_ji_* is a judgment that expresses the relation between the weights *w_i_* and *w_j_*, making *a_ij_* = *w_i_*/*w_j_* and *a_ik_* = *w_i_*/*w_k_* then:

(11) $$\frac{w_{i}}{w_{j}}\cdot \frac{w_{j}}{w_{k}}=\frac{w_{i}}{w_{k}}=a_{ik}$$

This relation is provided by Saaty [14,22]. Hence, once we know all the preferences of one criterion/alternative *i* over the other *n-1* criteria/alternatives, it is possible to establish a relationship to identify all the other preferences by the transitivity axiom. In the preference transitivity, if a DM prefers options *x* to *y* and *y* to *z,* he/she must prefer *x* to *z* [34]. In the AHP, where there is a preference scale, we can establish this relationship as, given a*ij* (w_i_/w_j_) and a*ik* (w_i_/w_k_), then a*jk* (w_j_/w_k_) is obtained by:

(12) $$\frac{w_{i}}{w_{j}}\frac{w_{i}}{w_{k}}=\frac{w_{j}}{w_{i}}\cdot \frac{w_{i}}{w_{k}}=\frac{w_{j}}{w_{k}}=a_{jk}$$

The expression shown in (12) enables identification of all other preference relations from a minimum (n-1) set of comparisons. The proposed method can be summarized in two steps:

1) Choose a criterion/alternative and perform all n-1 comparisons it demands;
2) Use expression (12) to obtain the other values from the matrix of pairwise comparisons.

To illustrate the method, consider a judgment matrix of four criteria as shown in Table 4, and the decision-maker chooses criterion 1 (c_1_) to compare with the others. In this case, the decision-maker makes the judgments of c_1_ with respect to c_2_, c_3_, and c_4_. From these initial judgments, other values are obtained.

**Table 4:** Four criteria matrix sample.

**Fig. 2:**
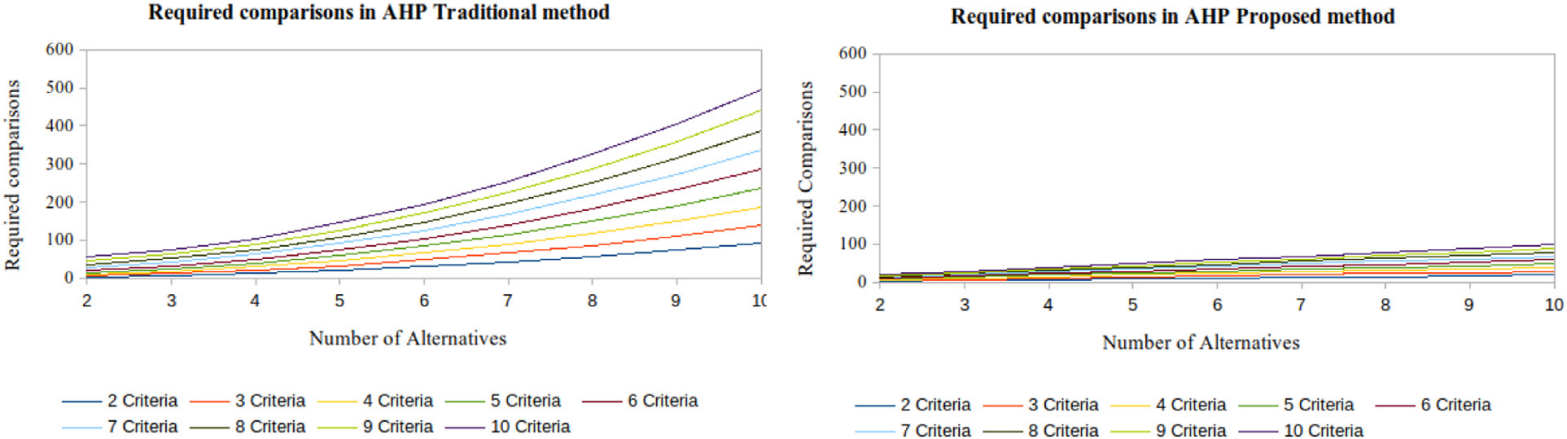
Number of judgments needed in the traditional AHP and in the proposed method.

This form allows all judgments at the same hierarchical level to be obtained from n-1 comparisons and with consistency value 0. Fig. 2 shows a comparison between the number of judgments needed in the traditional AHP and in the proposed method.

If the decision problem has 10 criteria and 10 alternatives, 495 judgments are required in traditional AHP method, but only 99 in the proposed method. Considering 1 min for each judgment, this means saving six hours and 40 min of the DM's time.

Of course, it would be unrealistic to require these relations to hold in the general case, mainly because human judgments contain deviations, biases, and inconsistencies [14]. However, nothing prevents the DM from occasionally revising the judgments, including those obtained automatically. These revisions can result in a consciously inconsistent judgment that is not possible with the traditional method of comparisons in the AHP.

To use the proposed method, web-based software was developed using popular web development technologies including PHP, HTML, CSS, JavaScript, Laravel framework, and MySQL database. The developed system allows group decision-making, considering the available IR values in [23], and the priority vector is obtained by the power method described earlier in this study. Each logical functionality of the application was tested by comparing the results with those expected in related literature. The system is available free of charge at https://www.ahpweb.net, and the source code is available at https://github.com/rvfrancozo/Simple-AHP-WEB.

## Results

### Structuring the decision problem

**Fig. 3:**
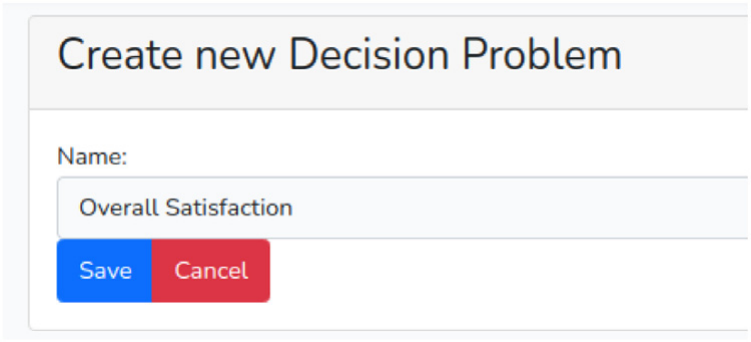
Create new decision problem.

The system is available at https://www.ahpweb.net, the user can access the system via social login with Google or GitHub or through a short user registration form. Next, the user clicks on “New Decision Problem” (Fig. 3), where he/she can enter the name of the decision problem or goal and save. As an example, we will create the European city choice problem available at [9, p. 4–9].

**Fig. 4:**
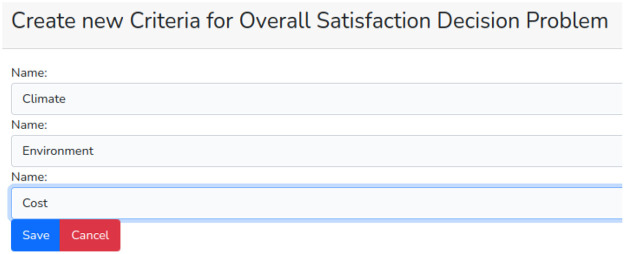
Create new criteria.

When creating a decision problem, the user is redirected to the list of problems, which is also accessible from the “My Decision Problems” menu. Two buttons are available next to the problem “Criteria” and “Alternatives”. First, the user must create the criteria, in this case: Climate, Environment and Cost (Fig. 4). The system allows the user to create up to 39 criteria; however, creating so many criteria or alternatives are not recommended.

**Fig. 5:**
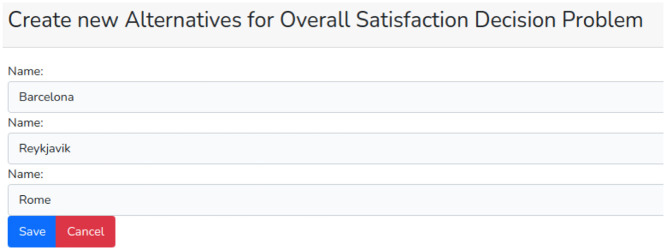
Create new alternatives.

After creating the criteria, the same procedure must be followed to create the alternatives, in this case: Barcelona, Reykjavik, and Rome (Fig. 5).

**Fig. 6:**
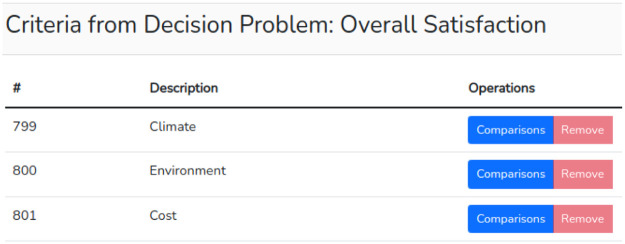
List of criteria.

After creating the criteria and alternatives, it is possible to proceed to the pairwise comparisons. In the problem list (accessible from the “My Decision Problems” menu), the user clicks on “criteria” to display the registered criteria and then chooses one of them to compare with the others as seen in Fig. 6.

**Fig. 7:**
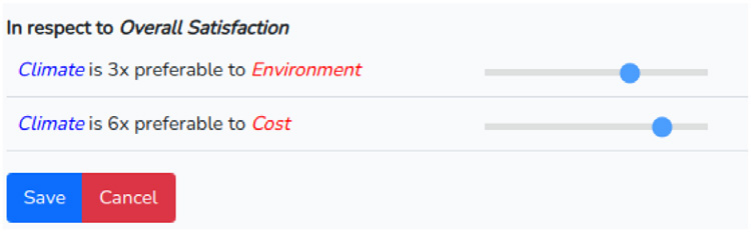
Intra criteria judgments.

Clicking on the “Comparisons” button of the chosen criterion will redirect the user to a page where he/she can make the “n-1″ comparisons needed. The slider can be moved to the right or left to assign the desired judgment. It is not necessary to do this for each criterion. In the example, we chose the criterion “Climate” and assigned the values 3 to the criterion “Environment” and 6 to “Cost” as in the example available in [9] and shown in Fig. 7.

**Fig. 8:**
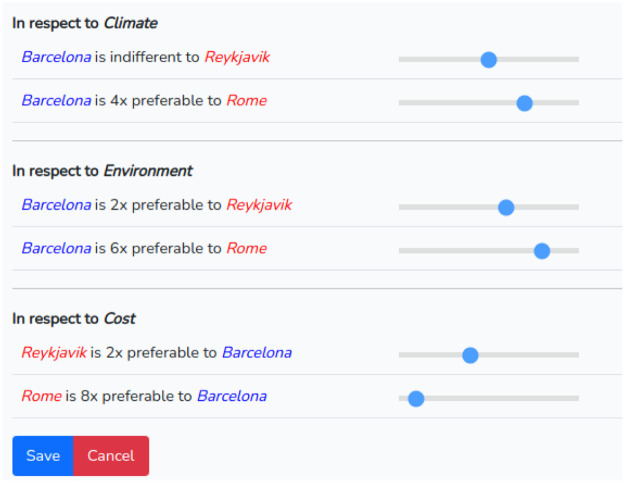
Intra alternative judgments.

A similar procedure should be performed for the judgment of the alternatives. The difference is that in this case the chosen alternative is compared with the others for each of the registered criteria. An example is given in Fig. 8.

**Fig. 9:**
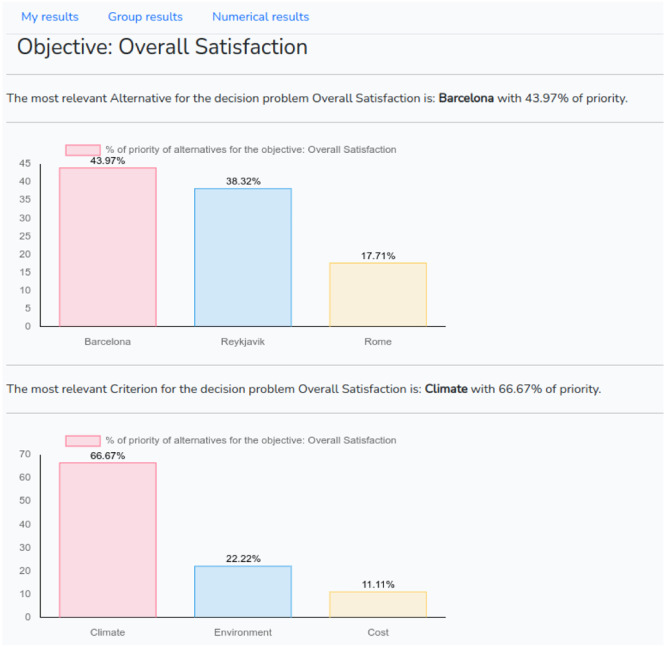
Graphical report.

After the judgments have been made, the user returns to the problem list and can click on the “Report” button to view the results, (Fig. 9) and make changes to the judgment.

The report page has three parts: “My results” shows the graphics with the results of the decision-maker's judgment for both criteria and alternatives. “Group results” shows the same as “My results” but aggregating the results of all the registered DM's. “Numerical results” show the comparison matrices. On this screen, the decision-maker can modify some judgment if he/she so wishes.

**Fig. 10:**
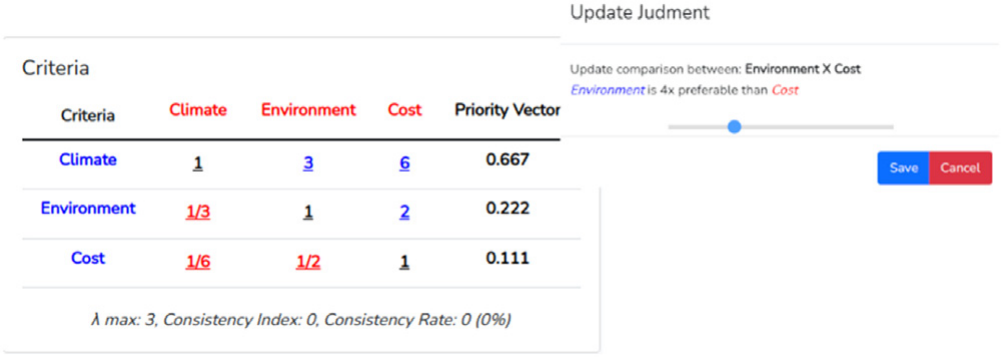
Updating a judgment.

In “Numerical results” to modify a judgment, the user simply clicks on a value within the matrix. The decision maker performs n-1 pairwise comparisons, the other comparisons are done automatically by the software so that the CR value equals 0. In Numerical results the decision maker can modify the automatically calculated judgments if desired. For example, the system calculated as 2 the importance of the criterion “Environment” over the criterion “Cost”, and this resulted in a CI = 0. When the user clicks on this value 2, the option to change the value appears. For example, let's set it to 4 (Fig. 10).

Now the value of λ_max_ is 3.054, CI = 0.027 and CR = 0.051.

### Group decision

The main differential of the proposed software is group decision. In the current version, the aggregation is done by the AIP method with geometric mean presented in the section Group Decision-Making. In future versions aggregation with AIJ method will be available.

**Fig. 11:**
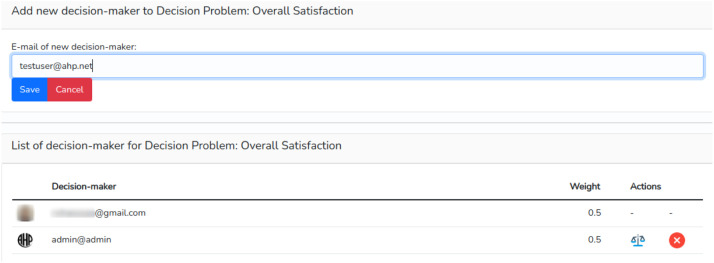
Adding new decision-maker.

To add DMs to the problem, it is necessary to go back to the problem list by clicking on “My Decision Problems” and click on the Group button. In the form that appears, simply enter the e-mail address of the new decision-maker as stated in Fig. 11.

Whenever a new DM is added, the weight of the decision is divided equally among all. The owner of the decision problem can click on the scale icon and perform a comparison between the registered decision-makers using Saaty's fundamental scale to define the relative importance of each DM over the others. To see the results, the user just needs to return to the report in the “Group results” menu.

The validation of the results was performed by comparing the results obtained by the software with those obtained in the literature in [6,9,37,38].

## Discussion

Various authors have discussed advantages and disadvantages of the AHP (References). In this study we focus specifically on three: the amount of pairwise comparisons in problems with a large number of criteria and/or alternatives; the consequent difficulty in maintaining consistency of judgments; and the absence of a software for group decision making. The software developed and presented in this study seeks an answer for these three issues.

The software is accessible via the web. It requires no installation and has no user requirements. The judgments are made interactively and require n-1 comparisons for the criteria and the alternatives. The decision maker still has the option to modify the entire judgment matrix and even consciously make an inconsistent judgment. Additionally it allows group decision making with the attribution of different weights for the decision makers.

The software presented here is an academic project in continuous development. As such, it has some important limitations. First, in the current version, it is not possible to add multiple levels of criteria and sub-criteria. Second, group decision is only available by aggregating individual priorities with geometric mean.

Even so, the software presents a differential in relation to the similar ones available, as shown in Table 5.

**Table 5:** AHP software comparison.

*Software Name*	*Free Version*	*Platform*	*Free Group Decision*
MakeItRational	no	Windows	no
Super Decisions	yes	Windows, Mac	no
Expert Choice	no	Windows	no
Logical Decisions	30 Day Trial	Windows	no
*AHP-WEB*	*yes*	*Independent*	*yes*

Currently the software has a base of over 100 active users with various decision problems such as: 2030 agenda; fire projects; ideal scenario for ethanol production; qualitative assessment of urban pedestrian crossings; rapid test for salmonella; prioritization of operational risk factors most relevant to the financial institution; criteria for population census among others. It is in use as a tool for further research in the context of performance indicators [35] and Small and Medium Enterprises [36].

## Conclusions

In this paper, a web-based expert system was developed to perform group or single decision-making with AHP. A web-based system requires no installations or specific operating system and can also be used remotely and on mobile devices. The developed system also allows the user to assign weights (or scale constants) to the DMs. Finally, the system takes advantage of the transitivity axiom to reduce the number of comparisons needed by DMs, which helps to reduce the time spent on analysis.

The system developed overcomes some of the main known limitations of software for AHP use and is an additional resource in the arsenal of AHP users. With the source code available in the git repository, any interested researcher can download, improve, and replicate the project within a controlled environment.

In future updates of the system, additional features are expected for sensitivity analysis, more levels of criteria/sub-criteria, implementation of the AHP with ratings and aggregation of individual judgments for group decision. Another important improvement to be made to the software in future versions is to perform formal unit tests, which are considered more robust and reliable because they ensure that the system follows the established standards and does not allow returns outside the specified rules. The current version is stable and well accepted by its users.

## CRediT authorship contribution statement

**Rafael Verão Françozo**: Conceptualization, Methodology, Software Development, Writing – original draft. **Luiz Sérgio Velasquez Urquiza Junior**: Software Development and Validation. **Elana Souza Carrapateira**: Software Development and Validation. **Bruna Cristine Scarduelli Pacheco**: Conceptualization, Visualization, Software. Writing – review & editing, Validation.

## Ethics statements

All authors declare that this work complies with ethical guidelines set by MethodsX.

## Declaration of Competing Interest

The authors declare that they have no known competing financial interests or personal relationships that could have appeared to influence the work reported in this paper.

## Data Availability

No data was used for the research described in the article.
